# Ultrafast low-pump fluence all-optical modulation based on graphene-metal hybrid metasurfaces

**DOI:** 10.1038/s41377-022-00787-8

**Published:** 2022-04-20

**Authors:** Ali Basiri, Md Zubair Ebne Rafique, Jing Bai, Shinhyuk Choi, Yu Yao

**Affiliations:** 1 School of Electrical, Computer and Energy Engineering, Arizona State University, Tempe, AZ USA; 2 Center for Photonic Innovation, Arizona State University, Tempe, AZ USA

**Keywords:** Optical techniques, Optical materials and structures

## Abstract

Graphene is an attractive material for all-optical modulation because of its ultrafast optical response and broad spectral coverage. However, all-optical graphene modulators reported so far require high pump fluence due to the ultrashort photo-carrier lifetime and limited absorption in graphene. We present modulator designs based on graphene-metal hybrid plasmonic metasurfaces with highly enhanced light-graphene interaction in the nanoscale hot spots at pump and probe (signal) wavelengths. Based on this design concept, we have demonstrated high-speed all-optical modulators at near and mid-infrared wavelengths (1.56 μm and above 6 μm) with significantly reduced pump fluence (1–2 orders of magnitude) and enhanced optical modulation. Ultrafast near-infrared pump-probe measurement results suggest that the modulators’ response times are ultimately determined by graphene’s ultrafast photocarrier relaxation times on the picosecond scale. The proposed designs hold the promise to address the challenges in the realization of ultrafast all-optical modulators for mid-and far-infrared wavelengths.

## Abstract (web summary)

Ultrafast low-pump fluence all-optical modulation for near and mid-infrared wavelengths is demonstrated by enhancing the interaction between graphene and pump/probe light in the nanoscale “hot spots” of graphene-plasmonic hybrid metasurfaces.

## Graphical abstract

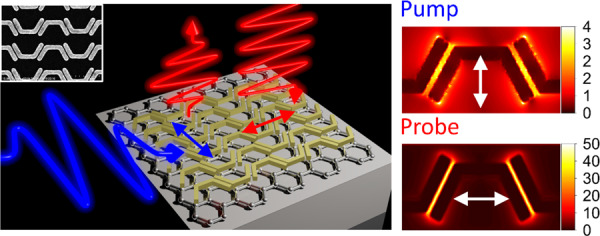

## Introduction

High-speed optical modulation is an essential ingredient for many applications, including optical interconnects^1^, ultrafast molecular spectroscopy^2^, material processing^3^, optical information processing and computation^4^, etc. All-optical modulation, compared to other techniques based on thermal, magnetic, acoustic, mechanical, and electrical effects, can achieve the highest modulation bandwidth up to THz. To date, all-optical modulation has been demonstrated based on a variety of materials and device designs, such as semiconductors waveguides^5^, colloidal plasmonic semiconductor nanocrystals^6^, chains of silicon nanoantennas^7^, gallium arsenide nanoparticles supporting Mie-type resonances^8^, nanostructured silicon membranes^9^, hot carrier effects of silver nanorods^10^, Tamm-plasmon resonance^11^, graphene-clad microfiber^12^, graphene–plasmonic slot waveguide^13^, thin-film absorber covered by graphene^14^, etc. Most of the modulators demonstrated experimentally are operated in the visible and near-infrared (IR) wavelength range. Ultrafast optical modulation for mid-and far-IR wavelengths is highly desirable for ultrafast molecular spectroscopy^15^, space communication^16–18^, remote sensing^19^, biomedical diagnostics^20–23^, and astronomical applications^24^. Researchers have explored all-optical modulators based on optically pumped sub-wavelength-structured optical membranes made of silicon^9^, broadly tunable plasmons in solution-processed, degenerately doped oxide nanoparticles^25^, two-photon absorption in germanium-on-silicon waveguides^5^, and bulk Dirac fermions in MBE-grown crystalline cadmium arsenide (Cd3As2)^26^ for wavelengths up to 6 µm. To date, it is still challenging to achieve ultrafast all-optical modulators for wavelengths >6 µm due to the inherent optical absorption and/or weak nonlinearity of optical materials.

Among all the materials studied for all-optical modulation so far, graphene holds the great promise to achieve ultrafast all-optical modulation over broad spectral regions from visible^27^ to terahertz (THz)^14,28^, due to its linear and gapless dispersion relation. Moreover, graphene possesses ultrafast carrier relaxation on a sub-picosecond time scale due to strong quantum confinement, enhanced carrier-carrier interaction, and the presence of massless Dirac fermions^29–32^. The state-of-the-art graphene-based optical modulators mainly fall into three major categories, i.e., electrically pumped modulators, thermo-optical modulators, and all-optical modulators. Electrically pumped graphene modulators have been demonstrated with high speed up to 35 GHz, limited by the RC constant of the external control circuit^33,34^. On the other hand, thermo-optical modulators have slower response times, i.e., usually a few hundreds of nanoseconds, fundamentally limited by the slow thermal diffusivity of most materials^29^. Finally, all-optical graphene modulators are the fastest, with picosecond response time^28,35,36^. One major challenge for graphene-based optical modulators is the limited absorption in the ultra-thin graphene layer and the resulted high pump fluence (~>300 μJ cm^−2^). Typical schemes to enhance the interaction between light and graphene include integration with dielectric waveguides^12^ and microfibers^35,36^, cavity^37^, and plasmonic slot waveguides^13^. Yet, it remains challenging to realize ultrafast all-optical modulation based on graphene with low pump fluences, especially for the mid-IR wavelengths.

This paper demonstrates ultrafast all-optical modulation based on subwavelength-thick graphene-plasmonic hybrid metasurface structures for near-IR and mid-IR wavelengths beyond 6μm. We integrated graphene with plasmonic metasurface absorbers to simultaneously enhance the light-graphene interaction at both the pump and probe wavelengths in the nanoscale plasmonic hotspots. The proposed device designs exhibit significantly improved all-optical modulation effects mainly due to two reasons. First, the pump light energy is focused onto nanoscale hotspots to generate a much larger photocarrier density than graphene devices without metasurfaces. Secondly, the device response at the probe wavelength is highly sensitive to the graphene optical properties in the hotspots and thus results in a strong modulation effect. Based on such design concepts, we realized all-optical modulators at both near-IR and mid-IR wavelengths with greatly reduced pump fluence (by 1–2 orders of magnitude) and enhanced optical modulation compared to purely graphene-based modulators. Furthermore, we performed near-IR ultrafast pump-probe measurement and proved that the proposed graphene-metal hybrid metasurface modulator design maintains the ultrafast response times of graphene-based modulators (on the picosecond scale), which is ultimately limited by the ultrashort photo-carrier relaxation times in graphene at room temperature (~2 ps for near IR and ~3 ps for mid-IR). The mid-IR all-optical optical modulator, to our best knowledge, is the first experimental demonstration of high-speed all-optical modulators for wavelengths beyond 6 µm with a pump fluence of <70 μJ cm^−2^. Similar design concepts are applicable for all-optical modulation for even longer wavelengths in the mid-to far-infrared spectral regions, for which it has been challenging to realize ultrafast optical modulators.

## Results

### Design concept

**Fig. 1:**
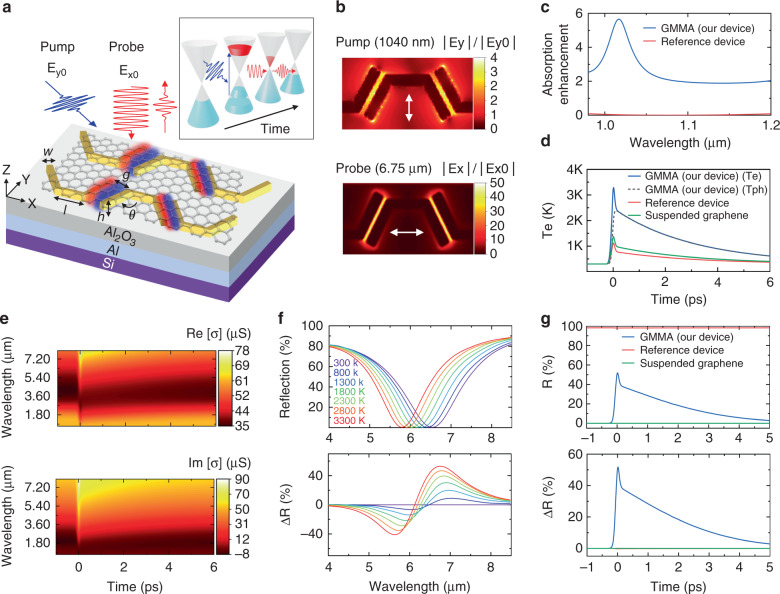
Design concept and theoretical modeling of the graphene-plasmonic hybrid metasurface all-optical modulator device design. **a** A schematic of a device with pump light (electric field vector along the Y-axis) and probe light (electric field vector along the X-axis) incident onto the surface, both focused into the nanoscale hot spots between closely coupled antennas. The inset illustrates the time-dependent interaction of pump and probe beams with graphene in the vicinity of the Dirac point. Here *l* = 340 nm, *w* = 80 nm, *h* = 40 nm, *g* = 25 nm, *θ* =120°. **b** Near-field enhancement in the graphene layer obtained by full-wave simulations for the pump at 1040 nm (top panel) and probe at ~6.5 µm (bottom panel). **c** The absorption enhancement in the graphene sheet located at the nanoscale hot spots of the GMMA devices (blue curve) and in the graphene of a reference device (red curve) with respect to that in a suspended graphene monolayer. **d** Simulated transient hot electron temperatures (T_e_, obtained with TTM method) of graphene in the GMMA device (blue curve, for graphene located in the nanoscale hot spots), the reference device (red curve) and a suspended graphene monolayer (green curve) as a result of the incident pump pulse (pulse duration: 100 fs; pulse fluence: ~70 μJ cm^−2^). The dashed black curve corresponds to the phonon temperature dynamics as a result of heat transfer from hot electrons and their subsequent equilibrium. **e** The real and imaginary parts of the graphene surface conductivity based on the random phase approximation (RPA) model, as functions of wavelength and time for the GMMA device for the same pump pulse as **d**. **f** Simulated reflection spectra (top) and differential reflection ΔR (bottom) of the GMMA device obtained by full-wave simulation, as functions of wavelength at various hot-electron temperatures from 300 K to 3300 K. Design parameters of the GMMA for this simulation are the same as those in **a**. **g** Simulated reflection (top panel) and differential reflection (bottom panel) of probe beam for the GMMA device (blue curve), the reference device (red curve) and a suspended graphene monolayer (green curve)

The proposed graphene-plasmonic hybrid metasurface device consists of a plasmonic metasurface, a graphene layer on top, a metallic back reflector, and a dielectric spacer layer (Fig. 1a). Closely coupled optical antennas form the plasmonic metasurface to maximize the interaction between light and monolayer graphene at both the pump and probe (signal) wavelengths. We utilized the metallic back reflector to form a tunable graphene-metallic metasurface absorber (GMMA) to enhance the optical modulation effect even further^38^. Figure 1b shows the near field intensity enhancement at 1.04 µm (pump, S-polarized, incident plane x-z) and 6.5 µm (probe, P-polarized) for a device design for operation wavelength around 6.5 µm. It clearly indicates that the near field intensity at the pump and probe wavelengths are significantly increased inside the nanogaps between the coupled nanoantennas, leading to strongly enhanced pump efficiency and reflection modulation. Full-wave simulation results show that the absorption in the graphene layer located in the nanogaps for the pump laser around 1040 nm is improved by about one order of magnitude compared with that of suspended monolayer graphene with ~2.3% absorption (Fig. 1c, blue curve). In comparison, the absorption in monolayer graphene on the spacer layer and back reflector without plasmonic antenna metasurfaces (used as a reference device in our experimental studies) is much lower than that in the proposed design (Fig. 1c, red curve). As a result, we expect a much larger photoexcited carrier population in the graphene layer close to the nanogaps than graphene without metasurfaces as in the reference device upon the incidence of pump light. Meanwhile, we designed the GMMA device with highly enhanced light-graphene interaction, esp. in the nanogaps, at probe (signal) wavelengths (Fig. 1b bottom panel). Thus, the optical response of the GMMA device is highly sensitive to the graphene layer inside and surrounding the nanogaps^38^. Note that, in the electrically controlled optical modulators presented in ref. ^38^, the graphene optical conductivity was tuned via changing the graphene Fermi level with a gate voltage, and the device design is mainly focused on realizing the broadest tuning range for mid-IR wavelength. For all-optical modulators here, we introduced transient changes of graphene optical property by creating photoexcited carriers with external laser pump pulses. The all-optical modulation design concept emphasizes the simultaneous enhancement for both the absorption of pump and probe laser beams at two different wavelengths in the same nanoscale ‘hot spot’ regions.

When the pump light is incident onto the device, the strong field enhancement inside the nanogaps between adjacent antennas leads to a large density of photo-carriers which quickly relax and recombine within a few picoseconds. As a result, the graphene inside the nanogap experiences a transient change in optical conductivity. Since the design also enables highly enhanced interaction between light and graphene inside the nanogaps between the coupled antennas at the mid-IR probe wavelength^38,39^, the transient change of graphene optical conductivity leads to a temporal change in the reflection spectra of the graphene metasurface absorber. We utilized a two-temperature model (TTM)^31^ and full-wave simulation to study the transient response of our devices. We adopted the TTM to simulate the dynamics of photocarriers generated by absorbed laser irradiance on a graphene sheet and their subsequent thermal relaxation to the optical (within hundreds of fs) and acoustic (within a few picoseconds) phonons^30,40^. For simplicity, the TTM method neglects the non-thermal photocarrier population in graphene, which decays much faster than the laser pulse duration in our measurements (~100 fs)^31^. Moreover, in this model, the hot carrier generation in plasmonic nanoantenna and its transport to graphene has been ignored, as it usually requires higher pump fluences than that used in our study to play a significant role^41–44^. More detailed information about the TTM and full-wave simulation is included in the Materials and Methods section. The absorption of the pump light in graphene increases the electron temperature from room temperature (~300 K) to a few thousand Kelvin^30,40^, as shown in Fig. 1d. Because of the localized surface plasmon resonance excitation in the gold nanoantenna and the subsequent large nearfield enhancement, the electron temperature modulation is significantly larger than that in the optically pumped graphene samples without the plasmonic antenna metasurface under the same pumping fluence. This, in turn, leads to highly enhanced optical tuning effects of the graphene surface conductivity. Figure 1e shows the real and imaginary components of graphene surface conductivity as a function of time and wavelength, obtained by random phase approximation (RPA) theory. The transient change of graphene optical conductivity in the mid-IR wavelength range has bi-exponential decay times <100 fs and ~3 ps, consistent with the experimental results (~100 fs and 3.4 ± 1 ps) obtained by ultrafast pump-probe spectroscopic measurements of epitaxial graphene^45–47^. As a result of the transient change of graphene optical conductivity, the optical resonance of the metasurface absorber is blue-shifted upon the incident of the fs-pump pulse and returns to its original spectral location after the photo-carriers relax to equilibrium at room temperature. Figure 1f shows the reflection spectra around the mid-IR probe wavelengths (top panel) for different hot electron temperatures, considering the entire unit cell. Without the pump light, there are no photo-carriers generated in graphene, and thus, the electron temperature is close to room temperature, the same as the graphene lattice temperature. The GMMA device exhibits high absorption/low reflection (R_off_) near its resonance wavelength (~6.5 μm) in the mid-IR region from 6 to 7 μm. As the pump pulses impinge on the device, the resonance wavelength is blue-shifted to around 5.8 μm, resulting in low absorption/high reflection (R_on_) from 6 to 7 μm. The differential reflection ΔR, defined as R_on_− R_off_, increases with larger resonance wavelength shift and/or lower reflection at “off” state (R_off_). By maximizing the interaction of probe and active layer (graphene), we achieve a larger shift of resonance and hence larger modulation of reflected light for a given number of generated photo-carriers. Figure 1f bottom panel shows the corresponding differential reflection at different peak electron temperatures as functions of wavelengths. Note that for shorter wavelengths from 5 to 6 μm, the device has high reflection without the pump light and low reflection with pump light, which results in a negative differential modulation, as shown in the bottom panel of Fig. 1f.

The response time of the proposed GMMA modulator is mainly determined by the ultrafast photocarrier dynamics in graphene. Figure 1g shows the time-dependent reflection for our GMMA device at the probe wavelength (~6.5 µm) obtained by full-wave simulation. Upon pump pulse incidence, the reflection of the GMMA modulator increases from ~1% (R_off_) to over 50% (R_on_) within 100 fs and returns to equilibrium with the biexponential decay time constants of ~38 fs and 2.8 ps, which are ultimately determined by the photo-generated hot carrier dynamics in graphene (Fig. 1d) and the resulted transient change of graphene optical conductivity in the mid-IR wavelengths (Fig. 1e). The corresponding modulation depth, defined as $\frac{{{{{\mathrm{R}}}}_{{{{\mathrm{on}}}}} - {{{\mathrm{R}}}}_{{{{\mathrm{off}}}}}}}{{{{{\mathrm{R}}}}_{{{{\mathrm{off}}}}}}}$, is estimated to be ~50 (or 5000% in percentage). As a comparison, neither the reference device (same as described in Fig. 1c) nor the suspended monolayer graphene show any noticeable change in their reflection under the same pump fluence, also shown in Fig. 1g. This comparison confirms that the strong modulation for the GMMA device indeed relies on the enhancement of light-graphene interaction achieved by the plasmonic antenna metasurfaces.

### Device fabrication and characterization

**Fig. 2:**
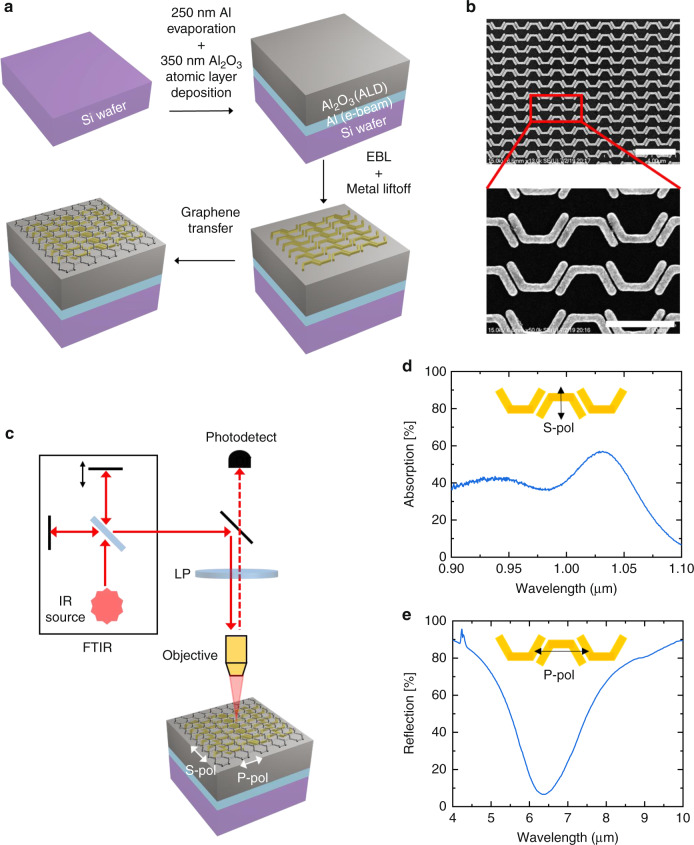
Fabrication and characterization of the GMMA all-optical modulator. **a** Fabrication steps of the GMMA device. **b** SEM images of fabricated Pi-shaped nanoantenna. The scale bars in the top and bottom panels represent 2 μm and 1 m, respectively. **c** A schematic of the reflection spectroscopy setup composed of an FTIR spectrometer and a mid-IR microscope. LP: linear polarizer. **d** The measured absorption spectrum of a GMMA device for s-polarized light around the pump wavelength at 1.04 μm (same design parameters as Fig. 1a). **e** Measured reflection spectra of the same GMMA device for p-polarized light with a resonance dip around 6.4 μm. The insets in d and e represent the polarization state of the incident light relative to the nanoantenna orientation

For experimental demonstrations, we fabricated the designed structures on silicon substrates. Figure 2 shows the major fabrication steps. First, 250 nm aluminum was deposited on a silicon wafer with electron beam evaporation as the metallic back reflector. Then a ~350 nm-thick layer of aluminum oxide was deposited with atomic layer deposition (ALD) to form the spacer layer. Next, the plasmonic antenna metasurface was patterned by electron beam lithography (EBL), metal evaporation (Cr~8 nm/Au~40 nm) and lift-off. Finally, a graphene monolayer was transferred on top of the plasmonic metasurfaces using a wet transfer process. The materials and methods section includes a more detailed description of the device fabrication process. Figure 2b shows the scanning electron microscope (SEM) images of the fabricated device before graphene transfer. The nanogaps between the adjacent antennas are measured to be about 29 nm with a standard deviation of about 3.9 nm (see Fig. S5 in the Supplementary Information).

We measured the reflection spectra of the fabricated devices using an FTIR spectrometer coupled to a mid-IR microscope. Figure 2c shows a schematic of the setup. Light from a broadband IR source passed a rotating linear polarizer (LP) to generate incident light with s-polarization or p-polarization. The reflected beam off the sample is collected by an objective lens and directed to a Mercury Cadmium Telluride (MCT) IR photodetector (see more details in the materials and methods section). Figure 2d shows the extracted absorption peak around the femtosecond pump laser wavelength at 1.04 µm for incident light with s-polarization. This corresponds to the total absorption in the graphene and plasmonic structure. We could not separately measure the absorption of the two materials in our experiment, as adding the graphene layer also affects the absorption in the plasmonic antennas as well the total absorption of the devices (Fig. S11). Yet, the absorption peak around the pump wavelength confirmed that the pump light was in resonance with our device, and thus its interaction with graphene was enhanced as expected in the simulation. As the nearfield simulation results (Fig. 1b) and absorption spectra (Fig. 1c) suggest, a significant fraction of the absorption is attributed to the absorption in the graphene at the hot spots in the antenna nanogaps. This feature enables our device to operate at ultra-low incident pump fluences. Figure 2e shows the reflection spectra in the mid-IR regime for incident light with p-polarization. The strong absorption around the resonance wavelength around 6.4 µm confirms highly enhanced light-matter interaction at the mid-IR probe (signal) wavelengths, which is essential for realizing highly enhanced modulation effects at low pump fluence. Compared with the simulation results, the resonance is blue-shifted by about 0.1 µm, which was likely due to the slight differences between the fabricated device dimensions and those used in the simulation.

### All-optical modulation at Mid-IR wavelengths

**Fig. 3:**
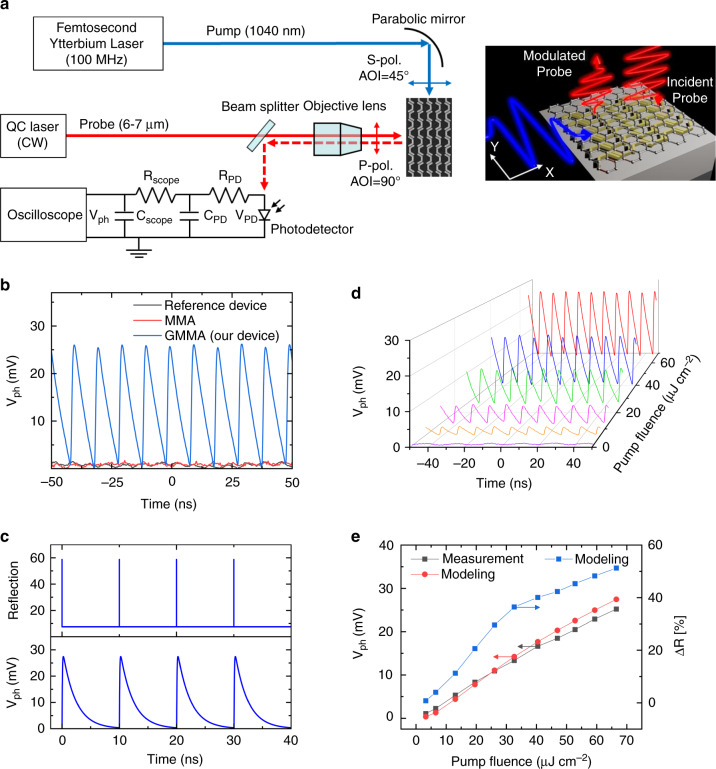
All-optical modulation for mid-IR wavelength. **a** The layout of the mid-IR modulation measurement setup. The inset shows a schematic of the GMMA mid-IR optical modulator with fs-laser near-IR pump, mid-IR continuous-wave probe (signal) and the modulated mid-IR pulse. **b** Measured detector photovoltage response to the modulated mid-IR laser beam output (at ~6.3 µm) from the proposed GMMA optical modulator (blue), in comparison with the reference device as presented in Fig. 1c (black) and an MMA structure without graphene (red). **c** Simulation results for the modulated reflection of a device at ~6.3 µm (top panel), and the simulated output photovoltage response of the mid-IR photodetector (bottom panel), taking the detector response time into account (≤10 ns). The simulation parameters (as defined in Fig. 1) are *l* = 340 nm, *w* = 80 nm, *h* = 40 nm, *g* = 25 nm, *θ* = 120°. **d** Measured detector photovoltage response to the modulated mid-IR laser beam output (at ~6.3 µm) from the GMMA optical modulator at different incident pump fluences. **e** Extracted average peak-to-peak detector photovoltage response V_ph_ (black squares) based on the measurement results in **d** and the simulation results (red circles) as a function of pump fluence. The plot also shows the corresponding simulated differential reflection ∆R (blue squares) achieved by the GMMA optical modulator (ΔR, defined as the maximum reflection change upon the incidence of the pump pulse)

For the experimental demonstration of mid-IR all-optical modulation, we used a femtosecond Ytterbium fiber laser at 1040 nm (pulse width ~100 fs, repetition rate 100 MHz) as the pump and a single-mode tunable continuous wave (CW) quantum cascade (QC) laser (wavelength tuning range: 5.97–6.92 µm) as the probe (signal). Figure 3a shows a schematic of the measurement setup. The s-polarized pump laser beam was focused by a parabolic mirror (NA = 0.125) and incident onto the device at an angle of 45°. The p-polarized probe laser beam passed a ZnSe objective (NA = 0.13) and incident vertically onto the device. The reflected probe beam from the device was then collected by a HgCdTe (MCT) fast photodetector (detector bandwidth = 500 MHz) by a parabolic mirror. We used a mixed domain oscilloscope with 16 GHz bandwidth to measure the output photovoltage response of the photodetector.

Figure 3b shows the measured photovoltage response on the MCT photodetector for a pump laser beam fluence of ~70 μJ cm^−2^. It is a pulse train with a repetition rate of 100 MHz and a pulse width of ~4.6 ns (FWHM). According to the theoretical model and simulation results presented in the design concept section and Fig. 1, the modulated mid-IR laser beam is a pulse train with the same repetition rate as the pump laser (100 MHz) and picosecond pulse width (Fig. 3c top panel), which is determined by the ultrafast photocarrier dynamics in graphene (Fig. 1d, g). However, due to the limited bandwidth of the photodetector (500 MHz) and oscilloscope (16 GHz), the photovoltage pulses measured by the oscilloscope are expected to be significantly broadened. Here, we modeled the limited bandwidth effects of photodetector and oscilloscope with two RC-integrator circuits in series (Fig. 3a), resulting in photovoltage pulse trains with a nanosecond pulse width (Fig. 3c, bottom panel) instead of the expected picosecond pulse width shown in the top panel of Fig. 3c. See the method section for more detailed descriptions of the calculation method. The FWHM of the pulses obtained in the simulation is ~1.8 ns, taking into account the limited bandwidth of the oscilloscope and photodetector. The measured FWHM of the pulses is ~4.65 ns. We attribute the broadened pulse width to reduced bandwidths of the measurement setup due to cables, adaptors, etc. Metals are known to have very large optical Kerr nonlinearity and hence are expected to play a role in the response of the device under high local field intensities. We also performed measurements on the reference device (as presented in Fig. 1b) and a metallic metasurface absorber (MMA) with the same design parameters as the GMMA device but without graphene. Neither shows any noticeable modulation at the same pumping level, as shown in Fig. 3b. Since the MMA has similar absorption at the pump and probe wavelengths as the GMMA design, the results also confirm that neither the thermally induced modulation nor the optical Kerr nonlinearity of gold plasmonic metasurfaces contributes noticeably to the modulation effects observed in our experiment due to the relatively low pump fluence used in our experiments (a few to a few tens of μJ cm^−2^). Usually, the large fluence is needed to produce nonlinear effects from plasmonic metasurfaces^48,49^. Therefore, we conclude that the pronounced mid-IR modulation of our device was mostly due to photocarrier generation in the graphene as well as the enhanced interactions with both pump and probe light in the GMMA device.

Next, we investigate the dependence of the reflection modulation on the pump laser fluence. We measured the modulated mid-IR laser beam with the MCT photodetector for different incident pump fluences. Figure 3d shows the detector photovoltage response to the modulated mid-IR laser beam by the GMMA optical modulator (same as in Fig. 3b) at 6.3 µm for pump fluences from 0 to 70 μJ cm^−2^. When there was no pump light on the sample (purple curve), no modulation was observed. Note that the weak and higher frequency oscillations in the absence of a pump laser were attributed to noise from the mid-IR laser probe source and elevated detector noise due to the input mid-IR laser beam. As we gradually increase the pump fluence, distinct modulation cycles start to show up at ~5 μJ cm^−2^ and continue to increase as the pump fluence increases. Figure 3e shows the extracted average peak-to-peak detector voltage response (V_ph_) as a function of the pump laser fluence on the device based on the measurement results in Fig. 3d. As a comparison, we also obtained the simulation results of differential reflection and the corresponding photovoltage response at the photodetector for different pump fluences, based on the simulated transient reflection in Fig. 3c. Both the measurement and simulation results suggest a monotonous increase of modulated pulse amplitude. The measured photovoltage shows slightly stronger sublinear behavior at higher pump fluences compared to simulation. In the current simulation, we ignored substrate heating effects, nonlinear effects in graphene, etc., which deserve a more in-depth study in future work. These results in Fig. 3d indicate that for 70 μJ cm^−2^ pump fluence, in principle and based on comparison with simulations, we expect the reflection can increase close to 50% (R_on_) at the peak pump power. When the pump is off, the measured reflection is about 6.4% (R_off_) at the probe wavelength). This corresponds to a modulation depth of ~680%.

The GMMA all-optical modulator design reduced the required pump fluence by about 1 to 2 orders of magnitude smaller than the closest competitive works in the literature for the mid-IR range (Fig. S10). Although our measurement results described above serve as a proof-of-concept demonstration for mid-IR modulation, we haven’t obtained the measured response time of the proposed device because the time resolution of the current measurement setup is limited by the mid-IR photodetector response time. According to theoretical modeling results presented in the design concept section, the response of GMMA mid-IR all-optical modulators has biexponential decay time constants of ~38 fs and 2.8 ps, fundamentally determined by the photocarrier dynamics in graphene and the resulted transient change of graphene optical conductivity in the mid-IR wavelengths. However, an ultrafast pump-probe measurement setup using phase-locked pump (near-IR) and probe (mid-IR) lasers with few tens of femtoseconds pulse widths and suppressed relative timing jitter was unavailable during our measurements. To experimentally investigate the response times of the proposed GMMA optical modulators, we further explored all-optical modulation based on the same double-enhanced GMMA device concept for near-IR wavelength around 1560 nm. We analyzed the near-IR femtosecond laser pump-probe measurement results to find out the fundamental limiting factors for the response times of GMMA devices, as presented in the following section.

### Ultrafast laser pump-probe measurement at near-IR wavelengths

**Fig. 4:**
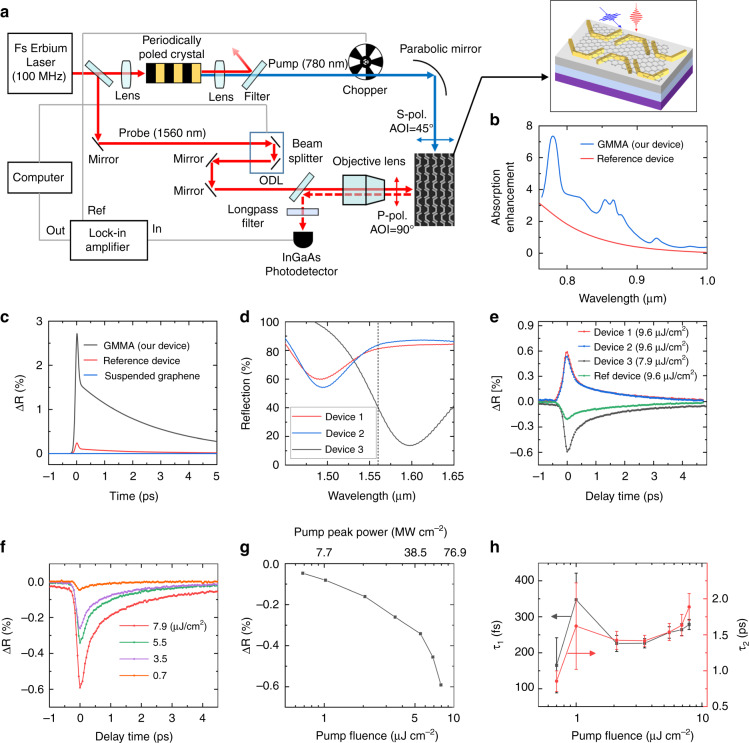
Ultrafast laser pump-probe measurement in the near-IR region. **a** A schematic of the pump-probe measurement setup. The inset shows a schematic of the GMMA modulator with fs-laser pump (blue) and probe (red) pulses. **b** The absorption enhancement in the graphene sheet located at the nanoscale hot spots of the GMMA devices (blue curve) and in the graphene of a reference device (red curve) with respect to that in a suspended graphene monolayer. The antenna length, width, gap and vertical periodicity of the plasmonic antenna metasurfaces are 360 nm, 100 nm, 30 nm, and 600 nm, respectively. **c** Simulated reflection modulation (∆R) for the GMMA device (black curve), the reference device (red curve) and a suspended graphene monolayer (blue curve) at different time delays for the probe wavelength at 1560 nm. **d** Measured reflection spectra of three GMMA devices with resonance at shorter (red and blue) and longer (black) wavelengths than the probe wavelength as indicated by the vertical dashed line. **e** The pump-probe differential reflection measurement results for the three devices in **d** and a reference device. **f** Measured differential reflection (ΔR) as a function of delay time at different pump fluences for GMMA device 3. The probe pulse fluence is ~2 nJ cm^−2^. **g** The maximal differential reflection as a function of incident pump fluences based on the pump-probe measurement results for GMMA device 3. **h** Experimentally extracted faster (left-axis) and slower (right-axis) bi-exponential decay time constants at different pump fluences for GMMA device 3

To investigate the fundamental limiting factors of the response time of the double-enhanced GMMA all-optical modulator design, we performed pump-probe measurements using a near-IR probe laser beam. The operation mechanism of the proposed GMMA modulator design also worked for the near IR probe around the wavelength of 1560 nm, even though the device design was not optimized for this wavelength range. Figure 4a shows a schematic of the measurement setup. The output from a femtosecond Erbium fiber laser (wavelength centered at 1560 nm) with 100 MHz repetition rate and ~100 fs pulse width was split into two beams. One laser beam was coupled into a periodically poled crystal to generate higher harmonics and subsequently filtered to only pass the second harmonic (SH) at 780 nm, used as the pump light. The other beam was directed to the optical delay line (ODL) to adjust its optical delay relative to the pump beam and used as the probe beam. Note that compared to the designed pump wavelength at 1040 nm (Fig. 1b, top panel), the pump wavelength at 780 nm corresponds to nearly the same near field enhancement inside the nanogap region (Fig. 4b). However, it provides a much higher time resolution in the pump-probe measurement than the Ytterbium laser at 1040 nm because of the excellent synchronization between the SH signal (780 nm) and the probe beam at 1560 nm. The pump laser beam is incident onto the sample at an oblique angle (45°, S-polarized), while the probe beam is vertically incident onto the same region on the device with P-polarization. The reflected probe light went through a long-pass filter (cutoff wavelength: 1150 nm and OD: 4) to filter out the scattered pump light and then was collected by an InGaAs photodetector (bandwidth 5 GHz). To improve the signal-to-noise ratio, we placed a chopper in the path of the pump beam and connected photodetector output to a lock-in amplifier to obtain the differential reflection signal for each time delay between the pump and probe pulses (see Materials and methods section for more details).

According to the simulation results (Fig. 4b), the absorption in the graphene layer in the nanogap “hot spot” of the GMMA device is enhanced by close to one order of magnitude compared to the suspended graphene monolayer. Similar to the mid-IR modulation scheme, this enhanced absorption results in a significantly increased change of the graphene optical surface conductivity upon arrival of the pump pulse, resulting in the blueshift of reflection spectra around the probe laser wavelength at 1560 nm (Fig. S8). We have performed theoretical calculations using the model as described in the design concept section and method section (numerical simulation). Here, the coupling of the optical phonons to the phonon bath at room temperature is assumed ~1.7 ps, extracted from near-IR pump-probe measurements. The pump pulse width and fluence used for the simulations were ~100 fs and 9.6 μJ cm^−2^, respectively. More details are presented in the materials and methods section. Figure 4c shows the reflection modulation at *λ*_0 _= 1.56 μm (i.e., ∆R = R(*λ*_0_, T_e_)–R(*λ*_0_, T_e_ = 300 K)). The initial raising time of differential reflection due to the photo-generated hot carriers can be modeled by a Boltzmann function producing a sigmoidal curve with ~70 fs time constant. The timescale of this behavior is below the time resolution of our system (~100 fs), limited by the pulse width of the pump and probe beams. Shortly after the pump onset, the reflectivity gradually returns to equilibrium state via hot carrier thermalization and the subsequent bi-exponential emission of optical and acoustic phonons on a timescale of ~240 fs and 1.7 ps. The maximum reflection modulation ∆R is ~3%, which is much smaller than that obtained for the mid-IR wavelength because of two major reasons. First, the change in optical conductivity of graphene for near IR wavelengths upon the incidence of the pump pulse is much smaller than mid-IR wavelengths (Fig. S3). Secondly, our modulator design was primarily optimized for the mid-IR range but not for the near-IR wavelengths. We compared the modulation results for three devices, i.e., the double-enhanced GMMA device design, a suspended graphene monolayer and a reference device (same as described in Fig. 1c). The GMMA device shows nearly one order of magnitude larger reflection modulation ∆R than the reference device and three orders of magnitude larger than the suspended graphene monolayer at the same pumping fluence.

Figure 4d, e shows the measured reflection spectra and the corresponding pump-probe measurement results of three devices. Devices 1 and 2 have resonance wavelengths around 1.5 μm (blue and red curves), which are shorter than the probe wavelength at 1560 nm. Device 3 has a resonance wavelength of around 1.6 μm (black curve), which is longer than the probe wavelength. Upon the incidence of a pump pulse, the reflection spectrum of GMMA devices is blue-shifted (Fig. S8); therefore, the wavelengths shorter than the resonance experience a negative differential reflection modulation, while the longer wavelengths exhibit a positive one. The measurement results in Fig. 4e show positive differential reflection for devices 1 and 2 and negative differential reflection for device 3 modulation signs, which agrees with the simulation results. Moreover, the GMMA devices show about three times larger modulation than the reference device due to the highly enhanced light-graphene interaction. Figure 4f shows the differential reflection pump-probe measurement results for device 3 at different pump fluences. When the pump fluence increased, more carriers were excited to the graphene conduction band. As a result, the maximal differential reflection increased as the pump fluence (Fig. 4g), which agrees reasonably well with simulation results (Fig. S9). We also extract the decay times of the pump-probe measurement results for different pump fluences. Figure 4h shows the obtained bi-exponential time constants as functions of pump fluence. The shorter time constant was around 200–300 fs and did not show a clear dependence on the pump fluence, which agrees reasonably well with our simulation results. The longer time constants were around 1–2 ps, slightly shorter than that of the reference device with only graphene (2.3 ps). This is likely due to the fact that the presence of gold nanoantenna opens up new relaxation channels for the optically excited hot carriers in graphene to relax towards an equilibrium state on a shorter time scale, which was not included in our theoretical model. This phenomenon is worth further investigation and could potentially be leveraged to reduce the response time of graphene-based devices.

The near-IR pump-probe measurements suggested that the modulation speed of all-optical GMMA modulators is mainly determined by the ultrafast photocarriers lifetime in the graphene. For mid-IR modulation, we expect the modulation speed to be fundamentally determined by the transient change of graphene optical conductivity upon incidence of fs-pump pulses. Since ultrafast pump-probe spectroscopic measurements in the literature reported a fast decay constant <100 fs and a slow decay constant ~3 ps for epitaxial graphene in mid-IR wavelength^45–47^, the mid-IR modulator response time should be on the scale of a few picoseconds, as we predicted based on the theoretical modeling results presented in Fig. 1g. The experimental proof of ultrafast response in the mid-IR regime will be pursued in future study.

## Discussion

In summary, we have demonstrated all-optical graphene-integrated metasurface modulators (GMMA) with subwavelength-thickness on silicon substrates, both at mid-IR (beyond 6 μm) and near-IR wavelengths based on a double-enhanced design concept with simultaneous enhancement of light-graphene interaction at both pump and probe (signal) wavelengths. The demonstrated mid-IR all-optical modulator was featured with greatly reduced pump-fluence and highly enhanced optical modulation depth at wavelengths above 6 μm. In comparison with other free-space ultrafast all-optical modulators reported in the literature (Fig. S10) based on different materials, such as conventional semiconductors composed of Si, Ge, III–V, and II–V (cadmium arsenide), plasmonic structures and thin-film oxides, the GMMA devices operate at one to two orders of magnitude lower pump fluence with comparable or larger modulation depth. The near-IR optical modulators (at 1.56 µm) showed three times higher reflection modulation than that of a reference device made of graphene on the same substrate without metasurface structures. Based on ultrafast near-IR pump-probe measurement, we obtained the response times of GMMA-based near-IR optical modulators in the range from 1 to 2 ps, slightly shorter than that of the reference device with only graphene (~2.3 ps). Both theoretical and measurement results suggested that the GMMA device design maintains the ultrafast response times of graphene-based modulators, which is ultimately limited by the transient change of graphene optical conductivity at the detector operation wavelength. In the mid-IR wavelength range, our theoretical model predicts the bi-exponential decay times of optically-modulated graphene optical conductivity to be <100 fs and ~3 ps, which is consistent with the experimental results (~100 fs and 3.4 ± 1 ps) obtained by ultrafast pump-probe spectroscopic measurements of epitaxial graphene^45–47^. Therefore, we expect the response time of the mid-IR GMMA all-optical modulators to be ~3 ps, limited by the longer decay time due to the phonon scattering process^30^. Our future efforts include conducting ultrafast pump-probe measurements with mid-IR femtosecond pulses as probe light to experimentally verify the optical response times of mid-IR modulation. Relying on the widely tunable optical conductivity of graphene as well as the broad resonance tunability of plasmonic antenna metasurfaces, the proposed GMMA device can potentially be a great candidate to fill out the technological gap of ultrafast all-optical modulators in the mid-to far-IR wavelength region with an ultra-compact footprint, ultrafast response times and record-low power requirements, which are deemed challenging in conventional modulator schemes.

## Materials and methods

### Numerical simulations

#### Two-temperature model (TTM)^40^ for graphene

In order to simulate the carrier dynamics in graphene and the consequent change in optical properties, we used the two-temperature model (it should be noted that this simplified model ignores the electron transport between the metal and graphene). In TTM, we consider excitations in the electronic system and in the strongly coupled optical phonons (SCOPs), each characterized by its respective temperature, T_el_ and T_op_, and linked by the electron-phonon coupling rate $\left( {{\Gamma}\left( {T_{{{{\mathrm{el}}}}},T_{{{{\mathrm{op}}}}}} \right)} \right)$:

1 $$\begin{array}{l}\frac{{dT_{el}\left( t \right)}}{{dt}} = \frac{{I\left( t \right) - {\Gamma}\left( {T_{el},T_{op}} \right)}}{{c_e\left( {T_{el}} \right)}}\\ \frac{{dT_{op}\left( t \right)}}{{dt}} = \frac{{{\Gamma}\left( {T_{el},T_{op}} \right)}}{{c_{op}\left( {T_{op}} \right)}} - \frac{{T_{op}(t) - T_0}}{{\tau _{op}}}\end{array}$$

In this description, the absorbed laser irradiance I(t) initially excites the electrons. The energy then flows into SCOPs at a rate described by:

2 $$\begin{array}{l}{\Gamma}\left( {T_{el},T_{op}} \right) = \beta \left( {1 + n\left( {T_{op}} \right){\int} {D\left( E \right)D\left( {E - \hbar {\Omega}} \right)f\left( {E,T_{el}} \right)\left( {1 - f\left( {E - \hbar {\Omega},T_{el}} \right)} \right)dE} } \right.\\ \left. {\quad \quad \quad \quad \quad - n\left( {T_{op}} \right)\mathop {\smallint }\nolimits^ D\left( E \right)D\left( {E + \hbar {\Omega}} \right)f\left( {E,T_{el}} \right)\left( {1 - f\left( {E + \hbar {\Omega},T_{el}} \right)} \right)dE} \right)\end{array}$$

This expression reflects the available phase space for electron scattering and includes only one adjustable parameter to describe the overall rate. Here *n*(*T*_*op*_) represents SCOP population, *f*(*E*, *T*_*el*_) id Fermi-Dirac distribution for electrons and $D\left( E \right) = \frac{{2E}}{{\pi \left( {\hbar v_F} \right)^2}}$ is the electron density of states in graphene. $\beta = 5eV^2cm^2s^{ - 1}$ is used for the best match with experiments. The specific heat of the electrons (*c*_*e*_) and the SCOPs (*c*_*op*_) are obtained, respectively, from theory and experimental data using Raman spectroscopy^40^. The slower coupling of the SCOPs to other phonons has also been included using relaxation time *τ*_*op*_ extracted from near-IR pump-probe measurements (~1.7 ps). We neglect the heating of these secondary phonons and assume that they remain at the ambient temperature of T_0 _= 300 K. The simulated behavior of maximum electron temperature (blue) and SCOP temperature (black) are plotted in Fig. 1d.

The elevated electron temperature leads to a change in graphene optical conductivity. The graphene optical conductivity can be modeled within the validity range of random phase approximation (RPA):

3 $$\begin{array}{l}\sigma \left( {\omega ,\gamma ,\mu _c,T_{el}} \right) = \sigma _{intra}\left( {\omega ,\gamma ,\mu _c,T_{el}} \right) + \sigma _{inter}\left( {\omega ,\gamma ,\mu _c,T_{el}} \right)\\ {\upsigma}_{intra}\left( {\omega ,\gamma ,\mu _c,T_{el}} \right) = \frac{{ - ie^2}}{{\pi \hbar ^2\left( {\omega + i2\gamma } \right)}}{\int}_0^\infty {E\left( {\frac{{\partial {{{\mathrm{f}}}}\left( E \right)}}{{\partial E}} - \frac{{\partial {{{\mathrm{f}}}}\left( { - E} \right)}}{{\partial E}}} \right){{{\mathrm{dE}}}}} \\ {\upsigma}_{{{{\mathrm{inter}}}}}\left( {\omega ,\gamma ,\mu _{{{\mathrm{c}}}},{{{\mathrm{T}}}}_{{{{\mathrm{el}}}}}} \right) = \frac{{{{{\mathrm{i}}}}\;{{{\mathrm{e}}}}^2\left( {\omega + {{{\mathrm{i}}}}2\gamma } \right)}}{{\pi \hbar ^2}}{\int}_0^\infty {\frac{{{{{\mathrm{f}}}}\left( { - {{{\mathrm{E}}}}} \right) - {{{\mathrm{f}}}}\left( {{{\mathrm{E}}}} \right)}}{{\left( {\omega + {{{\mathrm{i}}}}2\gamma } \right)^2 - 4\left( {{{{\mathrm{E}}}}/\hbar } \right)^2}}{{{\mathrm{dE}}}}} \end{array}$$

Here *γ* is the scattering rate of carriers, T_el_ is the electron temperature and μ_c_ the Fermi level. The optical response of such a graphene sheet can be modeled using a 2D surface conductivity in FDTD Lumerical Solutions to find the reflectivity of the device at each time step.

To model the ultrafast modulation behavior of our device, we assume the pump light is from a femtosecond laser. Figure 1d illustrates the photocarrier-induced dynamics before and after a pump pulse incident onto the device. Before the arrival of the optical excitation pulse, the carrier distribution is described by a Fermi–Dirac function at room temperature. The optical excitation generates a non-equilibrium distribution of hot electrons in the conduction band and holes in valence corresponding to an elevated electron temperature Te which can be as high as a few thousands of Kelvin^30,40^. Shortly after photocarrier generation, ultrafast Coulomb-induced carrier relaxation redistributes the excited carriers, and a hot Fermi–Dirac distribution is established via carrier–carrier interaction (Auger recombination and impact ionization) on a sub-100 fs time scale. This distribution cools down toward the lattice temperature via emission of optical phonons, super-collisions (impurity-assisted collisions) and interaction with acoustic phonons. Experimental studies of time- and angle-resolved photoemission spectroscopy in graphene suggest that the decay of electron temperature can be well described by a bi-exponential curve, corresponding to optical phonon emission (within a few hundreds of femtoseconds) and slower thermalization mechanism involving acoustic phonons (over a few picoseconds)^30^. This transient behavior of electron temperature under optical pulse excitation and its subsequent heat transfer to phonon modes can be well described using a two-temperature model (TTM)^31^ (see Fig. 1d).

Next, we applied the transient electron temperature values obtained by TTM into the FDTD simulator to find the corresponding reflection spectra. This enables us to simulate the change of reflectivity at the desired probe wavelength as a function of time. As the graphene electron temperature elevates from equilibrium, the metasurface resonance blue shifts due to local change of refractive index, resulting in an increase (decrease) in reflectivity of the device above (below) the original localized surface plasmon resonance of metasurface. Consequently, as the graphene electron temperature relaxes towards equilibrium, the relative reflectivity modulation returns to zero on the picoseconds time scale as well (Fig. 1g).

#### FDTD simulations

The FDTD simulations were performed using Lumerical Solutions FDTD. The material optical properties of gold, aluminum, aluminum oxide, and graphene are selected from the simulation package database. The thickness of each layer is determined by deposition rate and confirmed by a profilometer. The nanoantenna dimensions are extracted from SEM images. The plane wave source in the simulation is at normal incidence. We set the in-plane boundary conditions to periodic, with a perfectly matched layer (PML) and metal (also known as perfect electrical conductor (PEC)) for top and bottom out-of-plane (along the Z-axis in Fig. 1a) boundaries, respectively. Moreover, the nearfields for pump and probe wavelengths are resonantly enhanced in the nanogaps between the adjacent plasmonic nanoantennas and their surrounding regions. Therefore, in our simulations, we consider the elevation of electron temperature in the hot spot regions surrounding the nanogaps (nearly 10% of the unit cell area) and keep the electron temperature in equilibrium with room temperature everywhere else. The reflection spectra are then calculated based on the entire unit cell area. We used refined mesh grids in the graphene and nanoantenna interface with a minimum mesh size of 2.5 nm. The auto-shutoff for convergence of simulations was set to 5–10. The graphene scattering rate is 0.02 eV, and its chemical potential is 0.1 eV.

#### Calculation method for absorption

We used transmission box approach to find the absorption in different parts of the device. For example, to calculate absorption in Au Metasurface, we enclose the whole Au Metasurface inside the transmission box. As the metasurface is a periodic structure in the X and Y directions, we extend the transmission box outside the FDTD simulation region in those directions. Besides, we need to make sure that none of the monitors of the transmission box goes into metals. To avoid overlap of Au Metasurface and transmission box surface, we created an artificial 1 nm gap in-between Au Metasurface and Graphene.

### Fabrication

#### Back-reflector and spacer layer deposition

A 250 nm Aluminum back-reflector was deposited using electron beam evaporation (PVD 75, Kurt J. Lesker Company®). Then 350 nm Aluminum oxide was deposited through atomic layer deposition (Cambridge Savannah ALD) to form the spacer layer between the top metasurface structure and the bottom metallic back-reflector.

#### Nanoantenna fabrication

The aluminum oxide substrate was spin-coated with double-layer poly(methyl methacrylate) (PMMA) (120 nm 495k followed by 50 nm 950k) and a very thin (~5–10 nm) thermally evaporated Cr layer for charge dissipation. Then the samples were exposed to electron beam lithography (EBL, JEOL JBX-6000FS) and developed in a mixture of methyl isobutyl ketone (MIBK) and isopropanol (IPA) with a mixing ratio of 1:3. The sample was cleaned by oxygen plasma (Plasma-Therm 790, 5 sccm O2 with 8 mTorr chamber pressure, 20 W) for 30 s to remove the residual PMMA on the exposed region. Then 40 nm gold was deposited by thermal evaporation (Edwards Auto 306). Next, the gold nanoantenna was lifted off by soaking the sample in acetone for six hours, followed by sonication for 30 s.

#### Graphene transfer

We put one drop of DI water on glass slides and placed the graphene sample (a thin copper foil covered by graphene on both sides) on it. Then spin-coated 495 K PMMA with 3000RPM for 30 s. Next, flip the sample over and put it on a glass slide with the backside (without PMMA protective layer) facing up. We then covered the edges of the sample to fix its position and completely etched the backside graphene with O_2_ plasma for 15 min. In the next step, we cut around four edges of the sample to remove the boundary graphene residues on copper and floated the sample on copper etchant (CuCl_2_/HCl) with PMMA side facing up. The sample was transferred with a SiO_2_ wafer to DI water for three times to clean the copper etchant residue. Finally, we could pick up the sample with the expected substrate from DI water, dry it with N_2_ gun, rinse it with acetone and IPA and then dry the sample again with a nitrogen gun.

### Measurement

#### FTIR reflection spectra measurements

The optical reflection measurements at normal incidence were performed using a Bruker Vertex 70 FTIR spectrometer connected to a Hyperion 2000 mid-IR microscope (Fig. 2c). For the measurements with S-and P-polarization, we used a linear polarizer in the optical path right before the sample under test to ensure linear polarization incidence. The reflected light was collected by a 15x objective lens with NA = 0.4 and measured by a photovoltaic MCT detector. All the reflection spectra are normalized with respect to that of the bare aluminum oxide substrate to eliminate the impact of the substrate.

#### VIS-NIR pump-probe

The demonstration of near-IR modulation was implemented via a pump-probe spectroscopy setup, as discussed in Fig. 4a. The probe pulse was generated by the fundamental frequency of a femtosecond Erbium fiber laser with 100 MHz repetition rate and ~100 fs pulse width at 1560 nm with FWHM ~10 nm (Menlo Systems), while the pump was delivered through the second harmonic generation (SHG) process at 780 nm with a maximum fluence of ~10 μJ cm^−2^. The fluence of the laser pulse is defined by the optical energy delivered per unit area. It uses a full-beam area focused on the device surface. The pump beam was incident at 45° to the sample, while the probe beam was incident at a normal angle. The reflected probe light was collected after beam size shrinking by a combination of two plano-convex lenses and then focused onto a 5 GHz InGaAs photodetector (Thorlabs Inc.) (responsivity ~1 A W^−1^ at 1560 nm, noise equivalent power (NEP) $< 2 \times 10^{ - 15}\,{{{\mathrm{W}}}}\;{{{\mathrm{Hz}}}}^{ - 1/2}$) to analyze the reflectivity change. On the other hand, a long pass filter with a cutoff at 1150 nm and OD~4 was used to filter out the partially scattered pump light, which otherwise would be collected by the photodetector and interfere with the probe pulse. Next, we applied a phase-sensitive detection approach by placing a chopper on the pump path (optimized chopping frequency~1190 Hz) and set it as the reference signal for the lock-in amplifier. The photodetector output fed the input channel of the lock-in amplifier, and the DC output was recorded by the computer before the optical delay line (ODL) moved to the xt step. We used the instrument control toolbox in MATLAB to automate and synchronize the ODL movements and recording of the source meter readings.

#### MIR-NIR pump-probe

To investigate the device performance in mid-IR, we used a femtosecond Ytterbium fiber laser (Menlo Systems) at 1040 nm with 100 MHz repetition ra and ~100 fs pulse width as pump and a CW quantum cascade laser (Daylight Solutions) operating between 6 and 7 μm as probe laser as discussed in Fig. 3a. The beam area on the sample is ~1.5e-4 and ~1.1e-4 cm^2^ for the pump and probe, respectively. The pump was incident at 45° with S-polarization, whereas the probe was P-polarized at normal and focused on the sample by a ZnSe objective with NA = 0.13. The reflected probe beam was focused to a HgCdTe fast photodetector (bandwidth of 500 MHz) (PVI-2TE-10.6, VIGO System S.A.) by a parabolic mirror (reflected focal length of 2 inches) and displayed by a mixed domain oscilloscope (DSA-X 91604 A, Agilent Technologies) with 16 GHz bandwidth.

## Supplementary information

Supplementary information
