# Supplementary material for: Ultrafast low-pump fluence all-optical modulation based on graphene-metal hybrid metasurfaces
Source: Light Sci Appl. 2022 Apr 20;11:102. doi: 10.1038/s41377-022-00787-8 (PMC9021307; doi:10.1038/s41377-022-00787-8)
Supplement: Supplementary file 1 — Supplementary information [file 41377_2022_787_MOESM1_ESM.docx]

**Supplementary Information for**

**Ultrafast Low-pump Fluence All-Optical Modulation Based on Graphene-Metal Hybrid Metasurfaces**

*Ali Basiri*^1,2^*, Md Zubair Ebne Rafique*^1,2^*, Jing Bai*^1,2^*, Shinhyuk Choi*^1,2^*, Yu Yao*^1,2*^

^1^School of Electrical, Computer and Energy Engineering, Arizona State University, Tempe, AZ, USA, 85281

^2^Center for Photonic Innovation, Arizona State University, Tempe, AZ, USA, 85281

*Corresponding author： [**yuyao@asu.edu**](mailto:yuyao@asu.edu)

Contents

[1. Graphene sheet characterization and integration with metasurface absorber 2](#_Toc99698510)

[1.1. Characterization of graphene quality with Raman spectroscopy 2](#_Toc99698511)

[1.2 Graphene integration with metasurface structure 3](#_Toc99698512)

[2. Dependence of graphene surface conductivity spectra on electronic temperature 4](#_Toc99698513)

[3. Dependence of metasurface resonance wavelength on the design parameters 6](#_Toc99698514)

[4. Fabrication of Pi-shaped nanoantennas: nanogap size and statistical distribution 7](#_Toc99698515)

[5. Mid-infrared modulation: estimation of modulation depth and insertion loss based on simulations 7](#_Toc99698516)

[6. Near-infrared modulation 8](#_Toc99698517)

[6.1 dependence of reflectivity modulation of probe fluence 8](#_Toc99698518)

[6.2 Simulated resonance blueshift due to elevated electronic temperature 8](#_Toc99698519)

[6.3 Simulated transient negative reflectivity modulation at various pump fluences 9](#_Toc99698520)

[7. Comparison with all other techniques for ultrafast All-optical modulation 9](#_Toc99698521)

[8. FTIR absorption spectra around pump wavelength before and after graphene transfer 10](#_Toc99698522)

[9. Calculation of absorption in the plasmonic antenna around the pump and probe wavelengths 11](#_Toc99698523)

[10. Simulation at different incident angle 12](#_Toc99698524)

[11. References 13](#_Toc99698525)

# Graphene sheet characterization and integration with metasurface absorber

## Characterization of graphene quality with Raman spectroscopy

**Figure S1.** **Raman spectra after graphene transfer**. A representative Raman spectrum of modulator device covered by monolayer graphene. The large ratio of 2D peak to the G peak indicates a high-quality monolayer of graphene.

## Graphene integration with metasurface structure


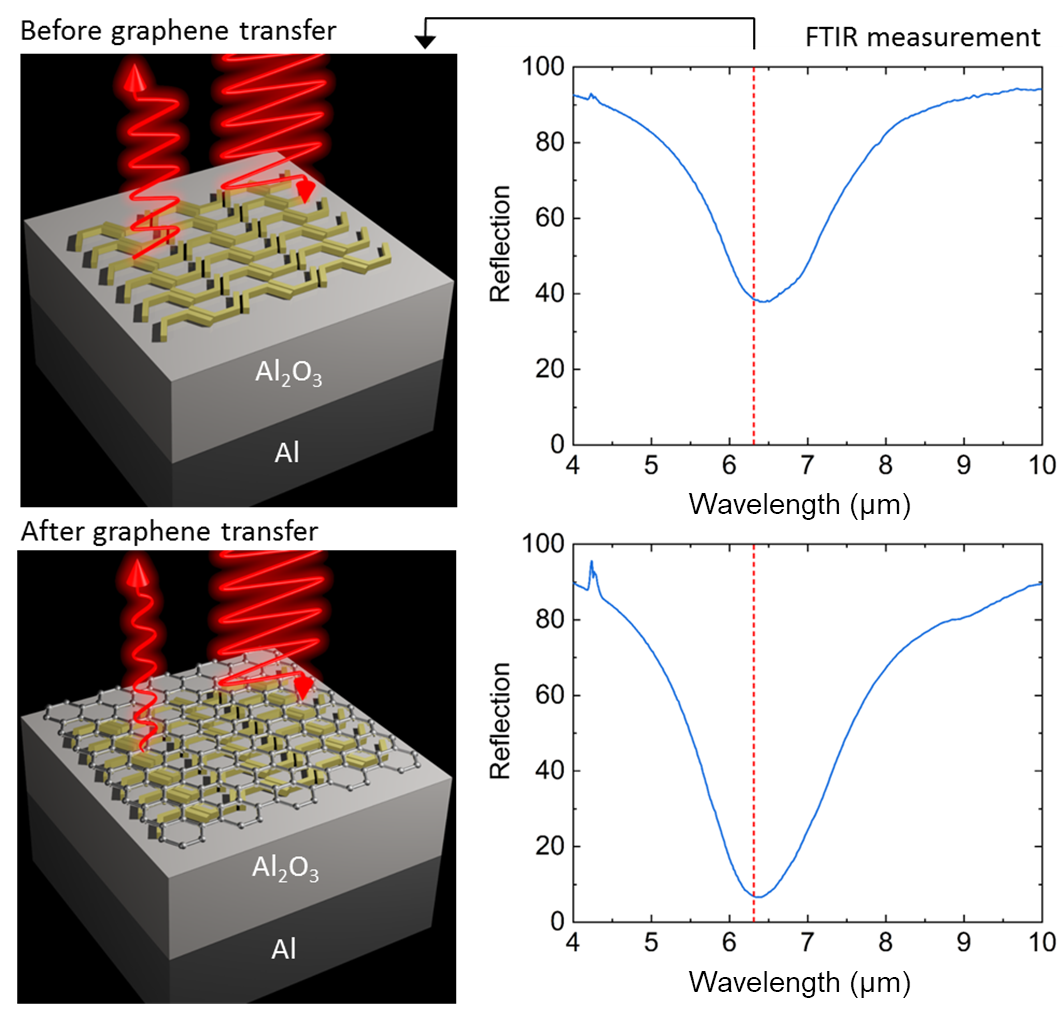


**Figure S2.** **Comparison of the device reflection spectra with and without graphene**. Reflection spectra before (top panel) and after (bottom panel) the graphene transfer. The schematics on the left column depict the change in amplitude of reflected beam at the desired operation wavelength of device, represented by red dashed lines on the right column.

# Dependence of graphene surface conductivity spectra on electronic temperature

The excitation of electrons in graphene from valence band to the conduction band is associated with the change of electric temperature and graphene surface conductivity. This process in turn alters the effective index of metasurface structure and therefore causes the blueshift of resonance within a sub-picosecond time scale. The change of real and imaginary components of the graphene surface conductivity are shown Figure S3


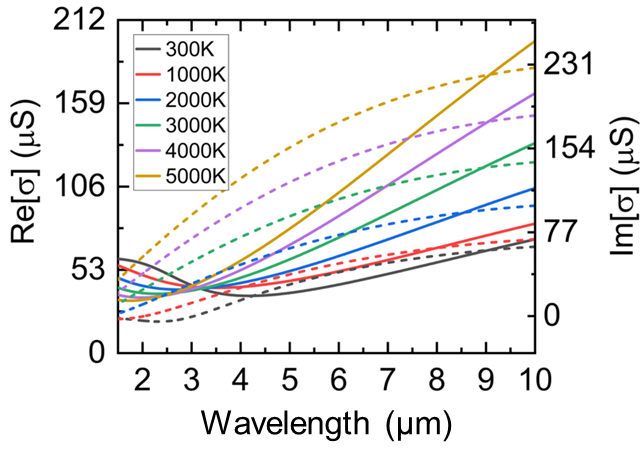


**Figure S3.** **Graphene surface conductivity vs wavelength at selected electronic temperatures**. Real (solid lines, left axis) and imaginary (dashed lines, right axis) components of the graphene surface conductivity over the near-IR and mid-IR wavelength range at various electronic temperatures (or equivalently time steps) as shown in Figure 1, part d and e.

# Dependence of metasurface resonance wavelength on the design parameters


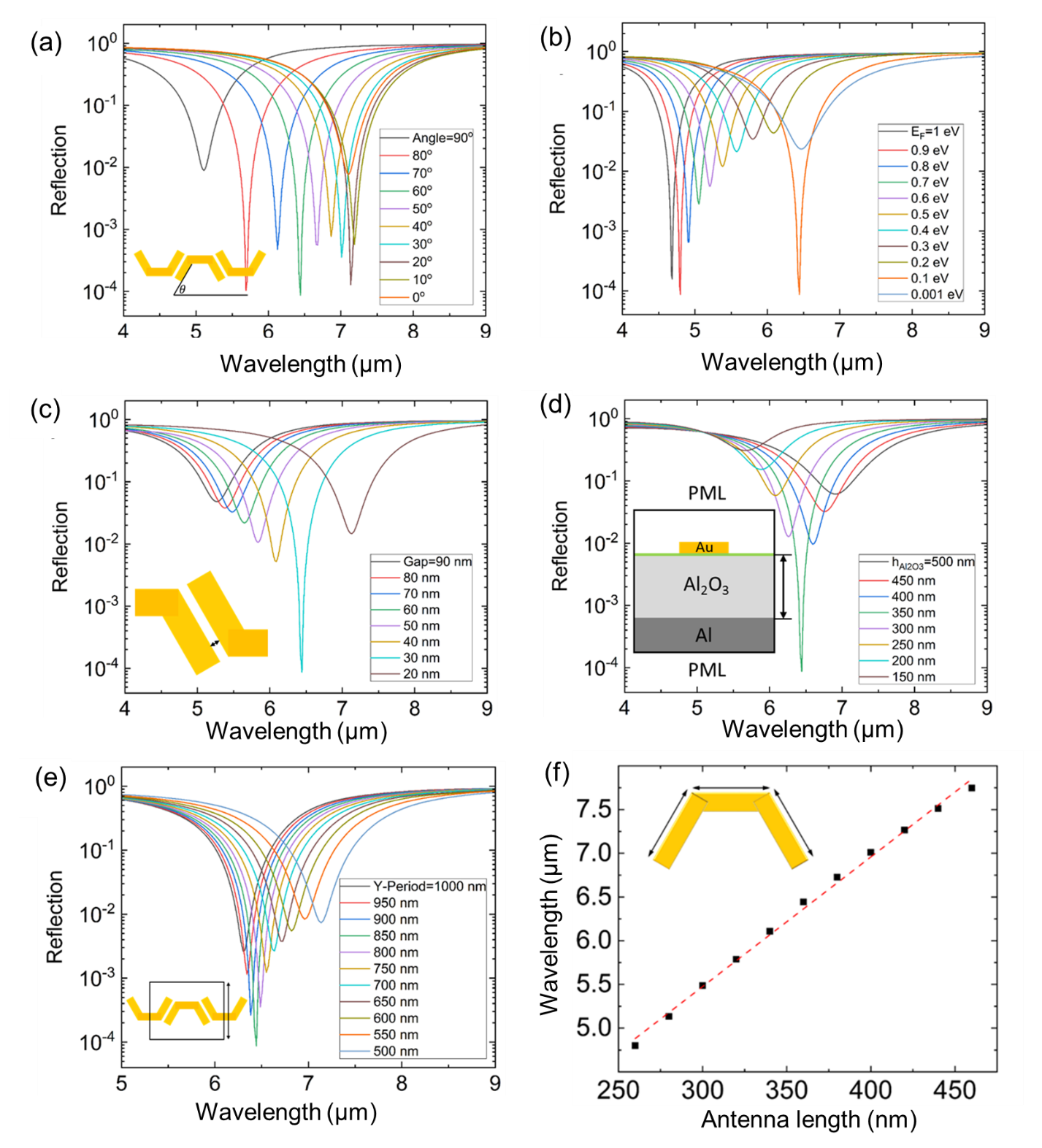


**Figure S4.** O**peration wavelength tunability of all-optical modulator**. FDTD simulations of device working wavelength as a function of various parameters.

# Fabrication of Pi-shaped nanoantennas: nanogap size and statistical distribution


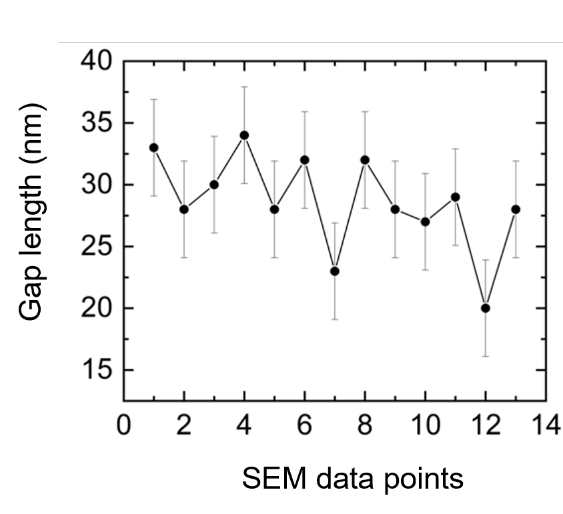


**Figure S5.** **Gap size variations base on SEM images**. Extracted nanogap lengths based on SEM images. The error bars indicate the corresponding standard deviation.

# Mid-infrared modulation: estimation of modulation depth and insertion loss based on simulations

**
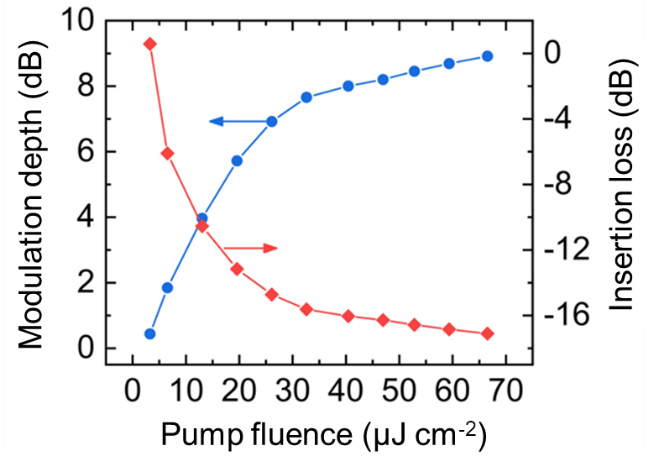
**

**Figure S6.** **Extracted modulation depth (in dB) and insertion loss from comparison of mid-IR measurements and numerical modeling**. Estimation of device modulation depth (blue color), defined as $10\times\log_{10} \frac{R_{\mathrm{on}}}{R_{\mathrm{on}}}$ in dB scale, and insertion loss (red color) obtained by comparison of average peak-to-peak voltage from oscilloscope waveforms at each pump fluence and numerical modeling of the device.

# Near-infrared modulation

## 6.1 dependence of reflectivity modulation of probe fluence


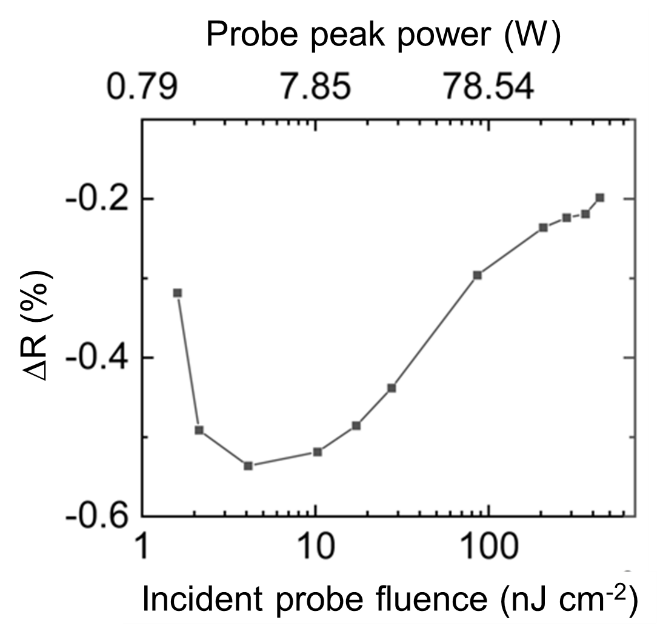


**Figure S7.** **Reflectivity modulation vs probe beam fluence**. Reflectivity modulation as a function of the incident probe fluence, illustrating the optimum required probe fluence to observe maximum reflectivity modulation.

## 6.2 Simulated resonance blueshift due to elevated electronic temperature

The simulated figure below shows that, as we expect, for a device with resonance wavelength (here around 1.48 µm) shorter than the probe wavelength, we expect a positive reflectivity modulation (shown by upward arrow). This prediction has been confirmed by measurements on devices 1 and 2 in the main text, Figure 4. In a similar vein, for a device with resonance wavelength longer than the probe wavelength, the reflectivity modulation will be negative (downward arrow in Figure S8), corresponding to device 3 in the main text, Figure 4.


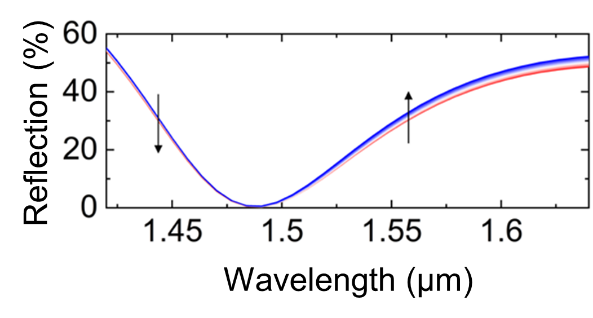


**Figure S8.** **Near-IR reflection spectra at different electronic temperatures**. Blueshift of reflection spectra by increasing the electronic temperature. The simulation parameters for nanoantenna are length=360 nm, width=100 nm, bending angle=60 degrees, nanogap size=30 nm, antenna thickness=40 nm.

## 6.3 Simulated transient negative reflectivity modulation at various pump fluences


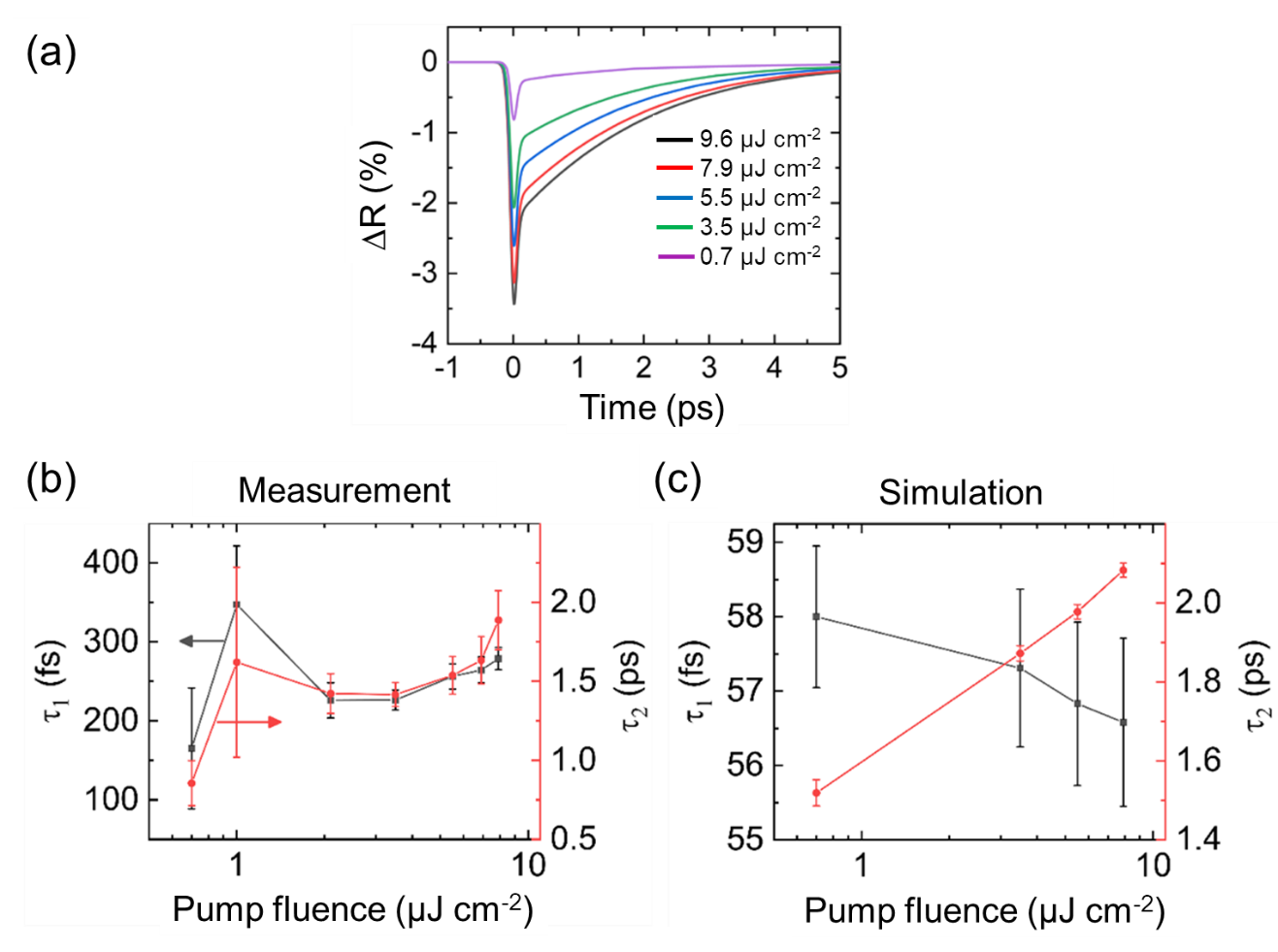


**Figure S9.** **Modulation depth at various pumping fluences vs delay time**. a) Simulated modulation depth at 1.56 $\mu m$, obtained by TTM and FDTD simulations. b) Bi-exponential decay rates extracted after fitting the transient pump-probe measurement data (left) and simulations (right) at various pump fluences.

# Comparison with all other techniques for ultrafast All-optical modulation

Figure S10 shows a variety of free-space modulators based on conventional semiconductors, plasmonic structures and thin-film oxides, in comparison to GMMA scheme. In each case, the pump fluence to achieve the best reported modulation depth has been considered. Our device exhibits a low pump fluence, high modulation depth, and large wavelength coverage, specially beyond 6 µm.

The change in surface conductivity of graphene is larger in mid-IR regime for the same amount of generated photocarriers (as shown in Figure S3). Therefore, for GMMA device one can expect a relatively lower fluence to achieve the same modulation at longer wavelengths. Compared to other devices, the required pump fluence is within 1-2 order of magnitude lower.

**
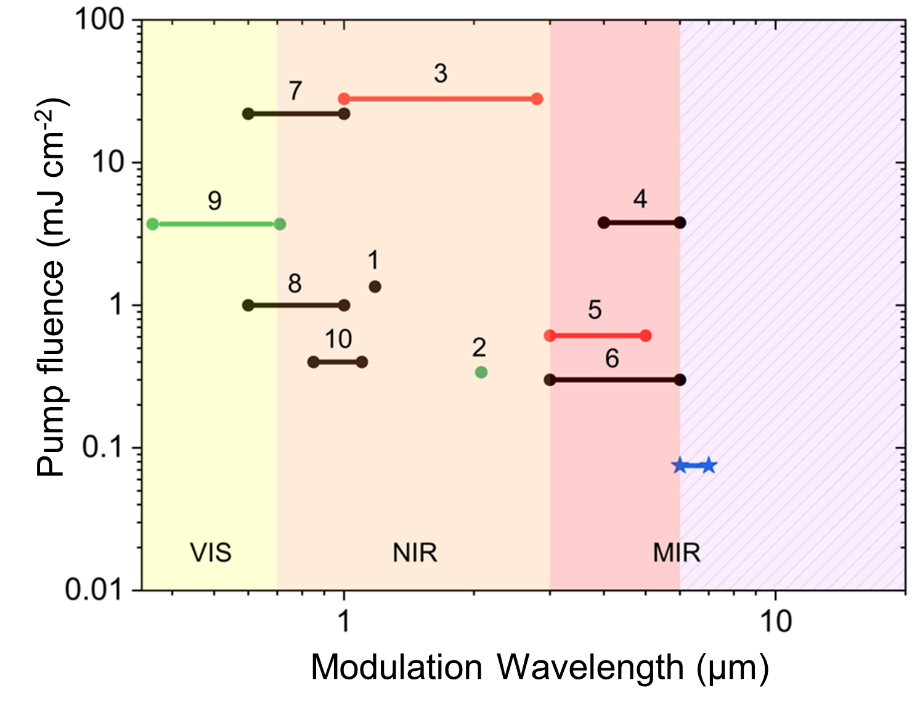
**

**Figure S10.** **Ultrafast all-optical modulator demonstrations**. Experimentally demonstrated ultrafast all-optical modulators. The vertical axis shows the required pump fluence of the modulator, while the horizontal axis indicates the modulation (probe) wavelength range. Different colors indicate different materials (black: conventional semiconductors including Si, Ge, III-V and II-V (cadmium arsenide); red: plasmonic; blue: graphene; green: thin-film oxides) in free-space^1-10^. The graphene-metal hybrid metasurface modulator (our work) is identified by blue stars. The modulation depth (defined by $\frac{\Delta R}{R}=\frac{R_{\mathrm{on}}{-R}_{\mathrm{off}}}{R_{\mathrm{off}}}$ or $\frac{\Delta T}{T}=\frac{T_{\mathrm{on}}{-T}_{\mathrm{off}}}{T_{\mathrm{off}}}$ ) for each reference in this figure is as follows: 20%^1^, 8530%^2^, 28%^3^, 80%^4^, 18%^5^, 9%^6^, 70%^7^, 37%^8^, 13%^9^, 50%^10^. In comparison, the extracted modulation depth ($\frac{\Delta R}{R})$ based on our measurements is ~680%.

# FTIR absorption spectra around pump wavelength before and after graphene transfer


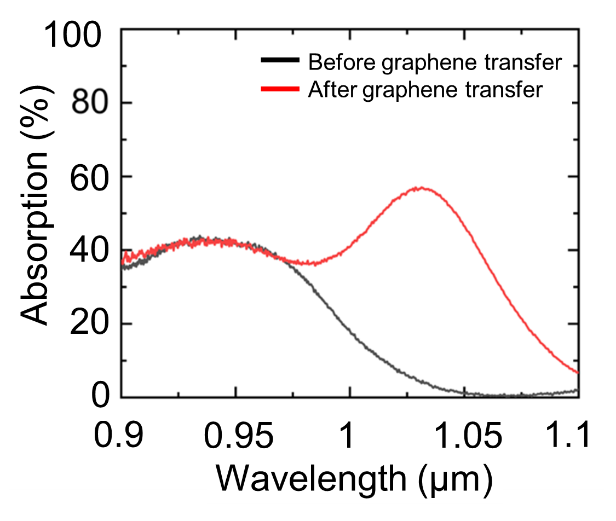


**Figure S11. FTIR absorption spectra.** The plot shows the total absorption spectra at normal incidence around the pump wavelength before (black) and after (red) the graphene transfer.

# Calculation of absorption in the plasmonic antenna around the pump and probe wavelengths

Pump light is incident on the device at an oblique angle (45 degree). Light absorption at 45˚ incident angle in different parts of the device (i.e., Au metasurface, graphene) w.r.t wavelength is provided in the Figure S12.


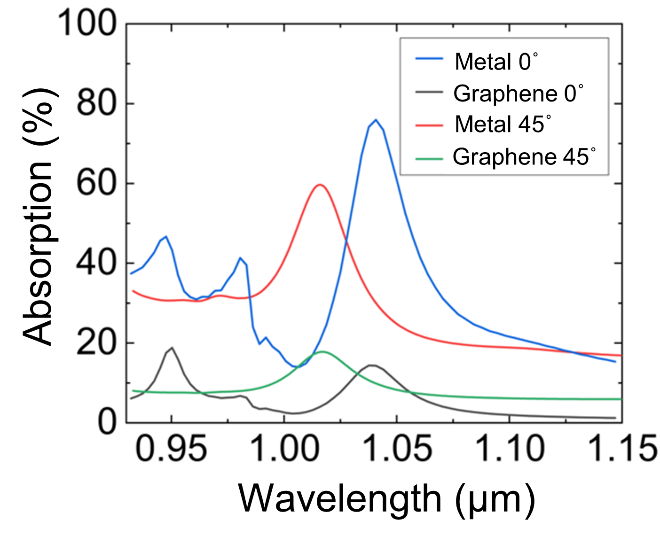


**Figure S12**. **Simulated absorption of light in different parts of the device in the NIR pump wavelength range.** S-polarized pump light is incident on the sample at 45˚. For the sake of comparison, we also included the absorption spectra under normal (0˚) angle of incidence. Note that the graphene absorption provided here was obtained in the whole graphene sheet while in Figure 1c we investigated the optical absorption enhancement in the graphene located at the hotspot regions near the nanogap between antennas to find out the increase of local photocarrier generation in the hot spots, which plays the major role in realizing spectral tuning and light modulation of the GMMA device.


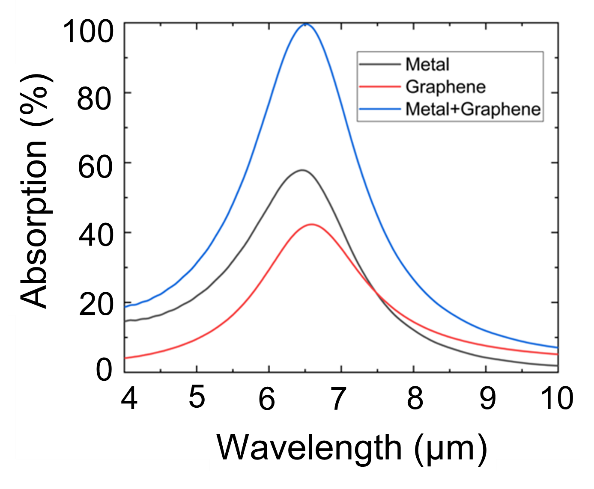


**Figure S13**: **Simulated absorption of light in MIR probe wavelength range.** The parameters used in this simulation are *l*=340 nm, *w*=80 nm, *h*=40 nm, *g*=25 nm, $\theta$=120⁰, Al_2_O_3_ thickness=350 nm. The electronic temperature in this simulation is 300 K.

Figure S13 shows the absorbed light in MIR wavelength range. The enhanced absorption in MIR helps to obtain enhanced reflection modulation (*R*_on_ - *R*_off_).

We use transmission box approach^11^ to find the absorption in different part of the device. For example, to calculate absorption in Au metasurface we enclose the whole Au metasurface inside the transmission box. As the metasurface is a periodic structure in X and Y direction, we extend the transmission box outside the FDTD simulation region in those directions. Also, we need to make sure that none of the monitors of the transmission box goes through metals. To avoid overlap of Au metasurface and transmission box surface, we create an artificial 1nm gap in-between Au metasurface and graphene.

# Simulation at different incident angle

The S-polarized pump light in our setup is incident on the sample at 45˚. The simulated reflection spectra of the device at 45˚ vs normal incidence are shown in Figure S14.


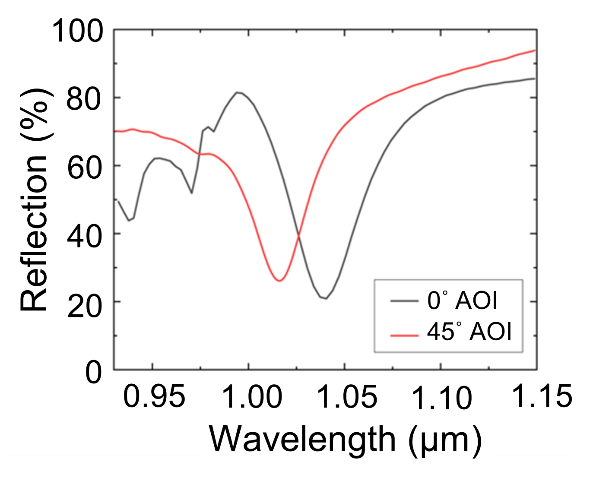


**Figure S14**: **Simulated reflection spectra of hybrid Metasurface for 45˚ and normal light angle of incident (AOI).** The parameters used in this simulation are l=360 nm, w=120 nm, h=40 nm, g=25 nm, $\theta$=120⁰, Al_2_O_3_ thickness=350 nm.

Our simulations suggest that under normal angle of incident (AOI) for pump (at 1040 nm), we expect nearly 20% reflection, while at 45˚ AOI reflection increases to about 65%. Although this deviates from the nominal design, one can always consider a slightly (~30 nm) redshifted nominal resonance under normal incidence to maximize the absorption at 45˚ AOI.

We also measured the reflected pump power at 45˚ AOI on the device and on the reflective unpatterned background and calculated the reflection to be between 60 to 70% for our fabricated devices, confirming the above trend observed in the simulations.

# References

1 Dani, K. M. *et al.* Subpicosecond Optical Switching with a Negative Index Metamaterial. *Nano Letters* **9**, 3565-3569, doi:10.1021/nl9017644 (2009).

2 Yang, Y. *et al.* Femtosecond optical polarization switching using a cadmium oxide-based perfect absorber. *Nature Photonics* **11**, 390-395, doi:10.1038/nphoton.2017.64 (2017).

3 Guo, Q. *et al.* Universal Near-Infrared and Mid-Infrared Optical Modulation for Ultrafast Pulse Generation Enabled by Colloidal Plasmonic Semiconductor Nanocrystals. *ACS Nano* **10**, 9463-9469, doi:10.1021/acsnano.6b04536 (2016).

4 Wu, R., Collins, J., Chekulaev, D. & Kaplan, A. All-Optical Modulation and Ultrafast Switching in MWIR with Sub-Wavelength Structured Silicon. *Applied Sciences* **9**, 1808 (2019).

5 Guo, Q. *et al.* Broadly Tunable Plasmons in Doped Oxide Nanoparticles for Ultrafast and Broadband Mid-Infrared All-Optical Switching. *ACS Nano* **12**, 12770-12777, doi:10.1021/acsnano.8b07866 (2018).

6 Zhu, C. *et al.* A robust and tuneable mid-infrared optical switch enabled by bulk Dirac fermions. *Nature Communications* **8**, 14111, doi:10.1038/ncomms14111 (2017).

7 Grinblat, G. *et al.* Ultrafast sub–30-fs all-optical switching based on gallium phosphide. *Science Advances* **5**, eaaw3262, doi:10.1126/sciadv.aaw3262 (2019).

8 Grinblat, G. *et al.* Efficient ultrafast all-optical modulation in a nonlinear crystalline gallium phosphide nanodisk at the anapole excitation. *Science Advances* **6**, eabb3123, doi:10.1126/sciadv.abb3123 (2020).

9 Guo, P. *et al.* Large optical nonlinearity of ITO nanorods for sub-picosecond all-optical modulation of the full-visible spectrum. *Nature Communications* **7**, 12892, doi:10.1038/ncomms12892 (2016).

10 Shcherbakov, M. R. *et al.* Ultrafast all-optical tuning of direct-gap semiconductor metasurfaces. *Nature Communications* **8**, 17, doi:10.1038/s41467-017-00019-3 (2017).

11 *Calculating absorbed optical power - Higher accuracy method with multiple materials*, <<https://support.lumerical.com/hc/en-us/articles/360034395294-Calculating-the-net-power-flow-with-a-Power-transmission-box>> (
